# siRNA-silencing of CD40 attenuates unilateral ureteral obstruction-induced kidney injury in mice

**DOI:** 10.1371/journal.pone.0215232

**Published:** 2019-04-12

**Authors:** Alonso Narváez, Roser Guiteras, Anna Sola, Anna Manonelles, Juan Morote, Juan Torras, Josep M. Grinyó, Josep M. Cruzado

**Affiliations:** 1 Experimental Nephrology, Department of Ciències Clíniques, Universitat de Barcelona, Institut d’Investigació Biomèdica de Bellvitge (IDIBELL), Hospitalet de Llobregat, Barcelona, Spain; 2 Department of Urology, Vall d’Hebron University Hospital, Barcelona, Spain; 3 Department of Nephrology, Bellvitge University Hospital, Barcelona, Spain; National Institutes of Health, UNITED STATES

## Abstract

**Background:**

The costimulatory CD40-CD40L pathway plays a role in kidney inflammation. We have previously reported that renal CD40 upregulation precedes cellular interstitial infiltrate and fibrosis in the unilateral ureteral obstruction (UUO) model. Here we sought to evaluate whether the administration of siRNA-CD40 has a therapeutic effect in a reversible unilateral ureteral obstruction (D-UUO) mice model.

**Methods:**

Eight week-old C57BL6J male mice were divided into four groups: Vehicle (Phosphate-buffered saline) (n = 8); siRNA SC (non-specific siRNA) (n = 6); siRNA-CD40 (n = 8) and WT (wild type) (n = 6) mice. UUO was performed with a microvascular clamp. At day 3 after surgery, the ureteral clamp was removed and nephrectomy of the contralateral kidney was performed. Immediately, PBS, siRNA SC (50μg) or siRNA-CD40 (50μg) was administrated via the tail vein. Mice were killed 48h hours after the siRNA or saline administration. Wild type (WT) mice were used as controls. Blood samples were collected for measuring creatinine and blood urea nitrogen (BUN). Histology and kidney mRNA expression were performed.

**Results:**

The administration of siRNA-CD40 reduced significantly the severity of acute renal failure associated with UUO. Pathologic analysis showed reduction of tubular dilation, interstitial fibrosis, F4/80 macrophage and CD3 (T cell) infiltration in animals treated with siRNA-CD40. Furthermore, kidney mRNA gene expression analysis showed significantly lower levels of macrophage markers (F4/80 and Mannose receptor), fibrosis matrix proteins (Fibronectin, MMP-9, Collagen IV and α-SMA), pro-inflammatory cytokines (iNOS and MCP-1) and the pro-fibrotic molecule TGF-β1 in siRNA-CD40.

**Conclusions:**

The administration of siRNA-CD40 therapy reduces the severity of the acute kidney injury induced by obstructive uropathy and promotes kidney repair. This strategy seems suitable to be tested in humans.

## Introduction

Obstructive nephropathy is a clinical syndrome resulting from structural and functional changes of urinary tract which is a common cause of Chronic kidney disease[1].

Renal interstitial fibrosis is the final pathway of obstructive nephropathy and is the major pathological basis studied[2]. Although not reversible at the late stage, renal interstitial fibrosis, which may have great significance in the prognosis of the disease, can be ameliorated and renal function could be improved provided with early and timely diagnosis and treatment[3].

The UUO (unilateral ureteral obstruction) model is the most classical used inducing renal fibrosis since most of them are irreversible[4]. However, the D-UUO (reversible unilateral ureteral obstruction) is a model that has been used to study the structural and functional recovery of the kidneys after relief of the obstruction and has much future potential for the study of inflammatory and immune processes, cellular and tissue regeneration because is a model similar to what occurs in the clinic[5,6]. But, only a few models have been described and the technique requires significant surgical expertise[7–9].

CD40 is a co-stimulatory molecule that belongs to the tumor necrosis factor superfamily. The CD40/CD40L dyad participates in T-cell proliferation and in effector functions[10]. It is expressed in many cell types, including epithelial tubular, endothelial, immune cells; and plays a role in kidney inflammation[11].

CD40-CD40L blockade using gene silencing strategies as a siRNA (small inhibitory RNA), have demonstrated its effectiveness therapeutic effects in several renal models: ischemia-reperfusion injury, acute allograft rejection, atherosclerosis, and autoimmune inflammatory processes[12–15]. Thus, CD40 has become a new emerging target[16].

Furthermore, because macrophages are recruited to local sites of the inflamed kidney and are critical during the inflammatory response, they are an ideal target for therapies[17]. In a previous study, our group reported that kidney pro-inflammatory genes such as CD40 were upregulated and precedes macrophage interstitial infiltrate and fibrosis in the UUO model[18].

In the present study, we hypothesized that blocking the co-stimulatory CD40-CD40L signaling by siRNA-CD40 (small inhibitory RNA anti-CD40) administration would reduce the inflammatory response and kidney damage in the obstructive nephropathy. Thus, in this study we sought to evaluate the therapeutic effect of siRNA-CD40 in kidney injury induced by obstructive nephropathy in a D-UUO mice model.

## Materials and methods

### Ethics statement and animals

Eight-weeks-old C57BL/6J male mice were purchased from Janvier (Laval, France), initial body weight of 21–26 g. Mice were monitored daily for body weight and were housed in groups of four per cage at constant temperature of 21 ± 2°C, with a 12 h-light/12 h-dark cycle and 55 ± 2% of humidity. They were given water and standard *chow ad libitum*.

All experiments were carried out in accordance with the Guidelines of the European Community Committee on Care and Use of Laboratory Animals and Good Laboratory Practice. The study complied with the current legislation on animal experiments in the European Union, and the principles of laboratory animal care were approved by the ethics committee for animal research of UB-Bellvitge (496/16).

### CD40-siRNA design

CD40 was silenced with a specific siRNA-CD40 in mice. Global patterns of expression of mRNAs in the obstructive kidney were compared among siRNA-CD40, siRNA SC (scrambled, non-specific siRNA), PBS (Vehicle) and wild type (WT) non-treated mice. The siRNA-CD40 sequence used in this study has been previously described by our group (siRNA TNFRSF5-3)[19]. It consists of a 21-nucleotides sense strand and antisense strand resulting in a two nucleotides overhang at the 3′ end of the antisense strand (sense 5′-GUGUGUUACGUGCAGUGACUU-3′, antisense 3′-GTCACACAAUGCACGUCACUG-5′). SC was used with a non-specific siRNA for off-target effects. The synthesis was carried out by an external manufacturer (Microsynth, Switzerland). To generate siRNAs from RNA single strands, equimolar amounts of complementary sense and antisense strands were mixed and 100% annealed. Oligonucleotides strands were annealed as previously described[15].

### Surgical technique and experimental design

**Fig 1:**
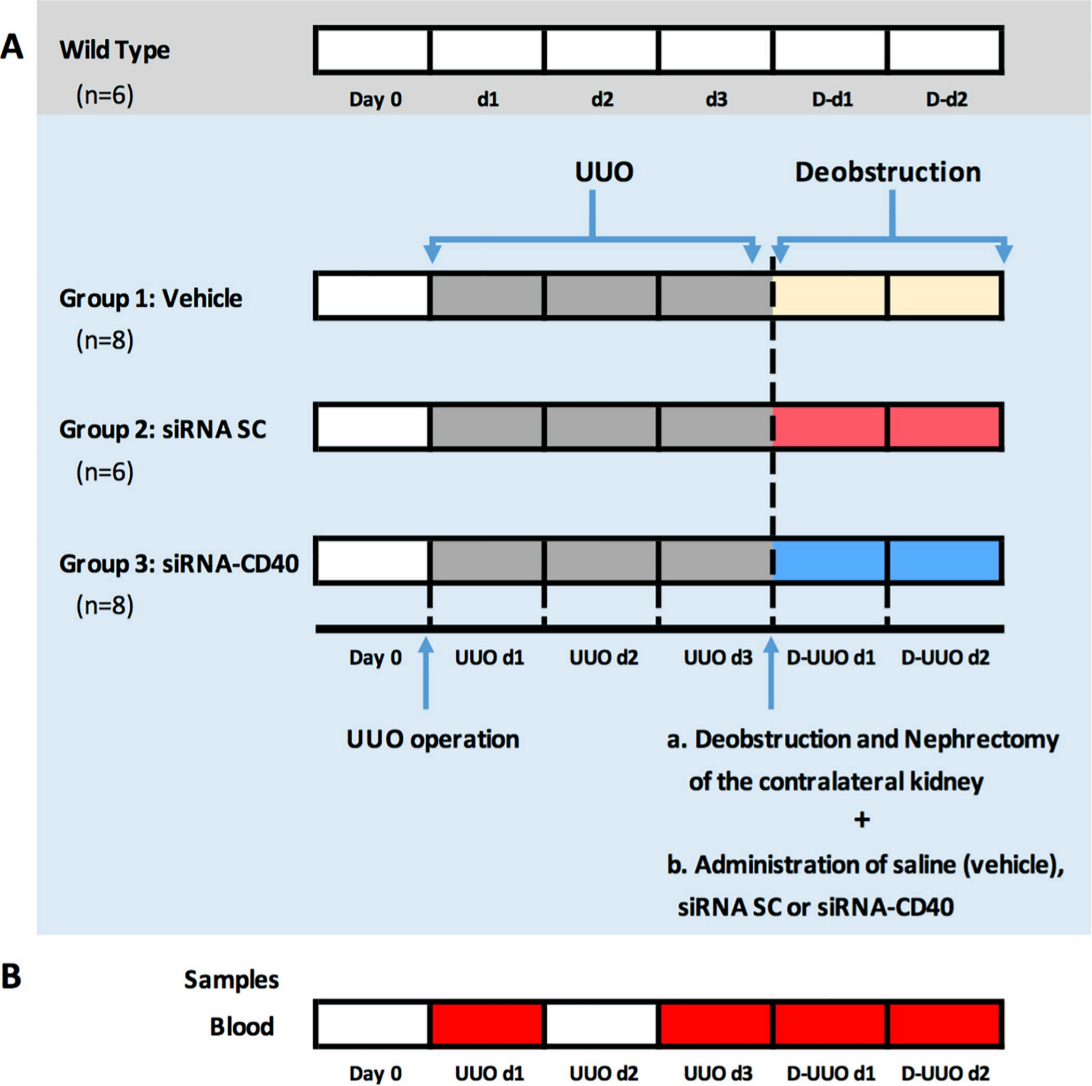
Scheme of the experimental design for the four groups of studied mice. **(A)** WT and groups 1 (Vehicle), group 2 (siRNA SC) and group 3 (siRNA CD40) where the D-UUO was performed with the administration of Vehicle, siRNA SC or siRNA CD40. (**B).** Blood samples were obtained at days UUO d1, UUO d3, D-UUO d1 and D-UUO d2.

C57BL/6J male mice were divided into four groups: Vehicle, animals with Phosphate-buffered saline (PBS) (n = 8); siRNA SC, animals with a non-specific siRNA (n = 6); siRNA-CD40, animals with specific siRNA anti-CD40 (n = 8) and WT, mice without any type of surgery or treatment administration served as control (n = 6). Fig 1 describes the study model with the following steps:

#### - UUO operation (UUO day 1)

UUO was performed under continuous inhalated isofluorane/oxygen anesthesia (2%). Mice received a pre-operative dose of analgesia (0.15 mg/kg buprenorfine, subcutaneously). The abdominal wall was opened doing a midline laparotomy and whereby the bowel gently displaced to one side. The right ureter was exposed and clamping with a one plastic microvascular clamp for vein vessel of 0.2–1 mm with atraumatic jaws (BIOVER). The bowel was then laid back in place and muscle and skin were then closed with sterile surgical of 4–0 nylon (B. Braun, Melsungen, Germany).

#### - Deobstruction, nephrectomy of the contralateral kidney and siRNA administration (UUO day 3)

At day 3 after clamping surgery, the ureteral clamp was removed and nephrectomy of the contralateral kidney was performed (D-UUO model). Immediately, PBS, 50μg of siRNA SC or siRNA-CD40 were administrated via the tail vein. The follow-up was 48 hours.

#### - Postoperative care

During the 6h postoperative period, normal activity (grooming, feeding, drinking, etc.) was monitored and the surgical wound and abdominal palpation were examined to rule out systemic infection. Mice tolerated the two surgeries without difficulty as evidenced by a rapid return to normal activity after recovery from anesthesia. There was no evidence of any adverse events.

#### - Samples

Renal function was assessed by measuring serum creatinine and blood urea nitrogen (BUN) following Jaffe’s and GLDH reactions (Olympus Autoanalyzer AU400, Hamburg, Germany) in the Veterinary Clinical Biochemistry Laboratory at Universitat Autònoma de Barcelona. Blood samples were extracted prior to UUO surgery (UUO d1), prior to deobstruction and nephrectomy (UUO d3), 24 hours (D-UUO d1) and 48 hours (D-UUO d2) after deobstruction and nephrectomy in all groups (Fig 1B). Blood was obtained on day 3 prior to surgery to avoid major aggression to the mice.

Mice were killed 48 hours after deobstruction and nephrectomy of the contralateral kidney under anesthesia via a heart puncture (blood collection). Kidney specimens were taken for histological and gene expression mRNA analysis.

### Optical microscopy, immunohistochemistry

Mice kidneys were removed under anesthesia and they were rapidly decapsulated and chopped into 2–3 mm small pieces. Renal slices were fixed in 10% (vol./vol.) formalin and embedded in paraffin. All pieces contained both cortex and medulla. Histological cross sections of 3μm thickness were stained with H&E (Haematoxylin & Eosin), Sirius Red, Masson Trichrome, α-SMA (1:100, Thermo Scientific, CA, USA,), F4/80 (1:50, LabClinics, Barcelona, Spain) and CD3 (1:150, Abcam, Cambridge, UK) for optical microscopy assessment. Histological sections were stained as described previously[20].

Sirius Red, Masson Trichrome, α-SMA, F4/80 and CD3 staining were quantified using Image J software in each non-overlapping cortical field from the cortical region. A magnification of 100x was used to evaluate the degree of renal injury. Red, brown and blue staining were considered as positive. Scores from 5 fields per kidney sections (10 per animal) were averaged and mean scores were quantified. Values are obtained as relative stained area (%). Pathological evaluation of tubular dilation was graded as follows: 0, no dilation; 1, changes affect <25%; 2, changes affect between 25–50% and 3, changes affect >50%. Cells located in the interstitial area were analyzed in 10 fields in each sample and assessed using Image J software. The percentage of CD3 positive cells was evaluated scoring total number of cells followed by manual counting of the number of CD3 positive cells per field. Immunostained sections without primary antibodies were used as negative control.

### Quantitative real-time PCR

RNA was extracted from kidney with PureLink RNA Mini Kit (Invitrogen, CA, USA) as previously described[20]. A total amount of 400 ng RNA was used to perform the reverse transcription using a High-Capacity cDNA Reverse Transcription Kit (Applied Biosystems, Warrington, UK). Thermal cycling conditions were 10 min at 25ºC, 120 min at 37ºC, 5 min at 85ºC and finally held at 4ºC. The mRNA expression levels of TGF-β1 (transforming growth factor- β1), CD40, MCP-1 (monocyte chemotactic protein-1), IL-2, Cytokeratin-18, iNOS (inducible nitric oxide synthase), Fibronectin, MMP-9 (Matrix metallopectidase 9), MR (Mannose receptor), F4/80, MCSF (Macrophage Colony Stimulating Factor), Collagen IV and α-SMA were quantified by TaqMan real-time PCR (ABI Prism 7700, Applied Biosystems, Waltham, USA) using the comparative 2^-(delta)(delta)Ct^ method (Applied Biosystems).

### Statistical analysis

All data are reported as mean ± SE. Group means were compared with either the Student’s *t* test or ANOVA for parametric values, or the Mann-Whitney *U* test or Krustal-Wallis test for non-parametric values. For semiquantitative variables the Chi-squared test was used. *P* ≤ 0.05 was considered to be statistically significant. All statistical analyses were carried out using StatView software.

## Results

### Acute kidney injury

**Fig 2:**
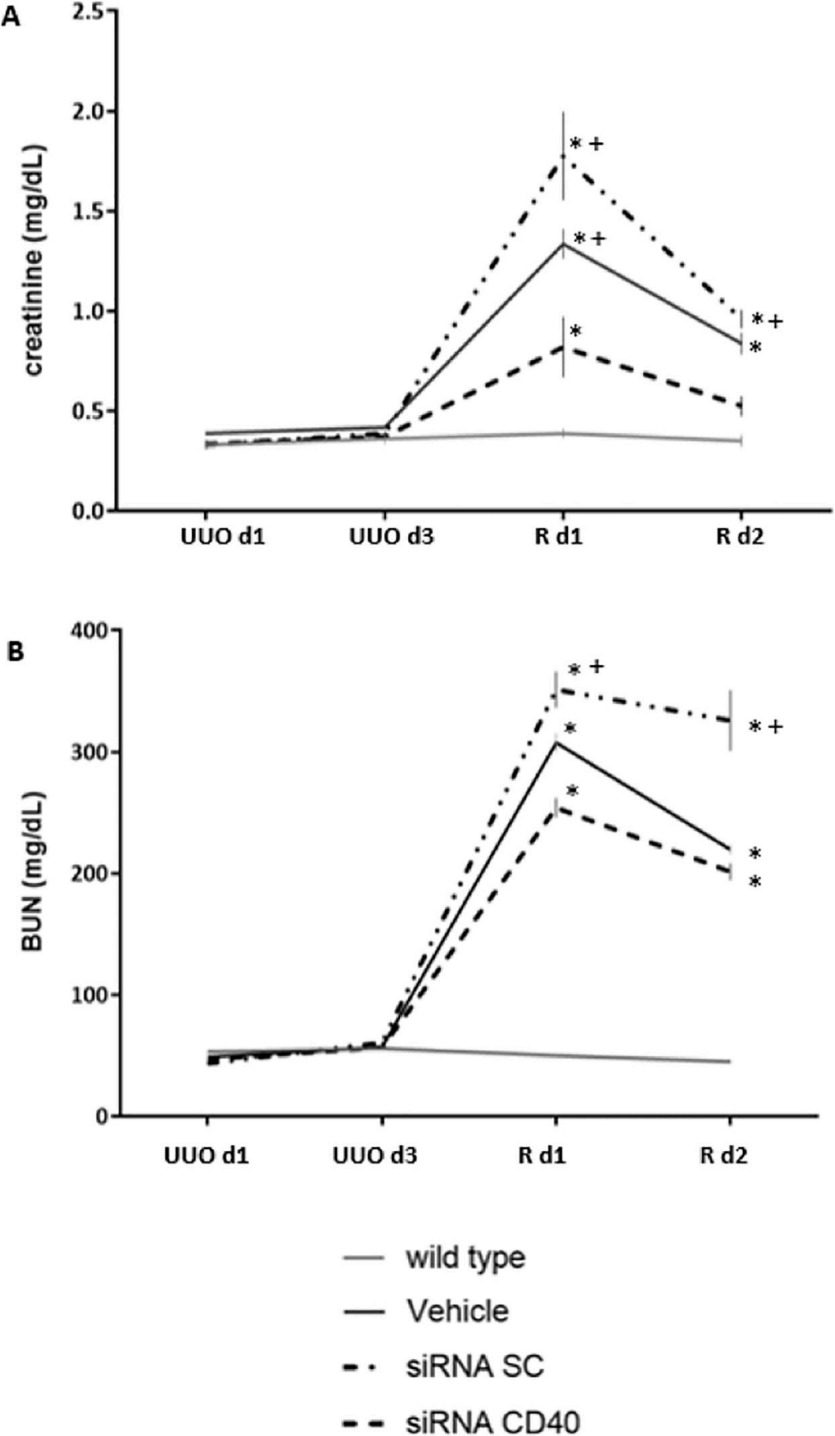
Renal Function analysis. **(A)** Creatinine and **(B)** Blood Urea Nitrogen (BUN) analysis. In all groups, both creatinine and BUN levels significantly increased showing maximum values at day 1 post clamp release (D-UUO d1) compared to the non-treated animals (WT). siRNA-CD40 treated group showed lower levels of creatinine and BUN reflecting a fast recovery of the renal function. ANOVA test; data are means ± SE, *p ≤ 0.05 vs WT, ^+^p ≤ 0.05 vs siRNA-CD40.

First, as seen in Fig 2, the siRNA SC and Vehicle groups showed worse renal function compared to siRNA CD40 treated mice. In all groups, there was evidence of acute kidney injury, with higher creatinine and BUN levels compared to its baseline values and reaching maximum values at day 1 after declamping and the nephrectomy of the contralateral kidney (D-UUO d1). Subsequently, creatinine levels decreased tending to normalization demonstrating the validity of the D-UUO model. BUN levels showed parallel results.

Second, siRNA-CD40 treated group showed significantly lower levels of creatinine and BUN compared to the others groups. Thus, siRNA-CD40 administration reduced significantly the severity of acute renal failure associated with UUO (Fig 2).

### Kidney tubular damage and inflammatory infiltration

#### Histology analysis

**Fig 3:**
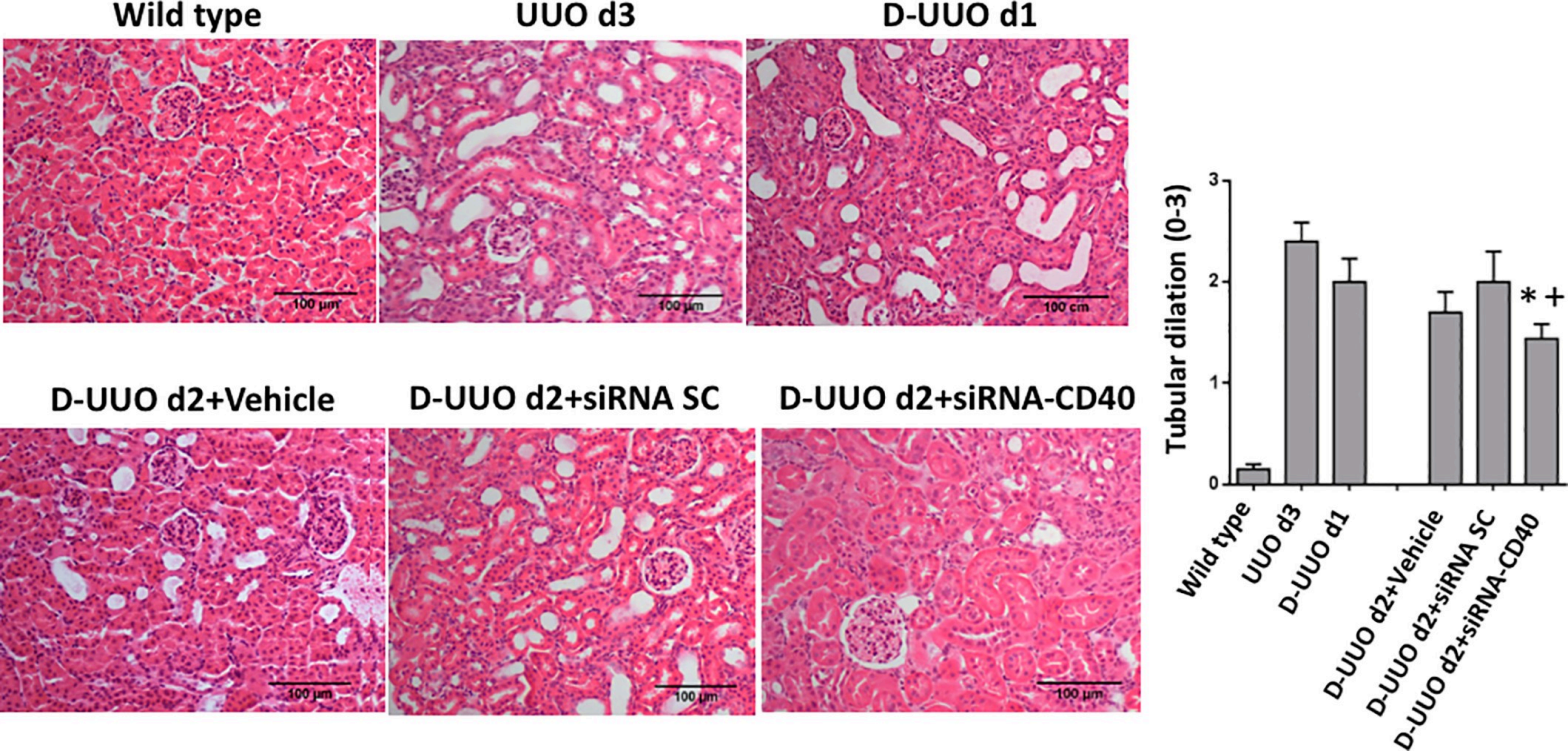
Tubulo-interstitial damage evaluation of the obstructed kidneys. Representative images of Haematoxylin & Eosin (H&E) staining. Original magnification 100x. Graphical quantification of H&E from each group using tubular dilation analysis. Chi-squared test; the values represent the mean ± SE, *p ≤ 0.05 vs D-UUO d2, ^+^p ≤ 0.05 vs D-UUO d2+siRNA SC.

At day 3 of obstruction (UUO d3), tubular dilation was clearly observed. Tubular epithelial cells showed vacuolar degeneration together with partial renal tubular lumen expansion. Once the clamp was removed, from day 1 to 2 after deobstruction (D-UUO d1 and D-UUO d2 respectively), pathological lesions were significantly ameliorated. Renal lesions caused by UUO and in particular tubular dilation were significantly attenuated at day 2 after deobstruction, especially in animals treated with siRNA-CD40 compared to others groups (Fig 3).

**Fig 4:**
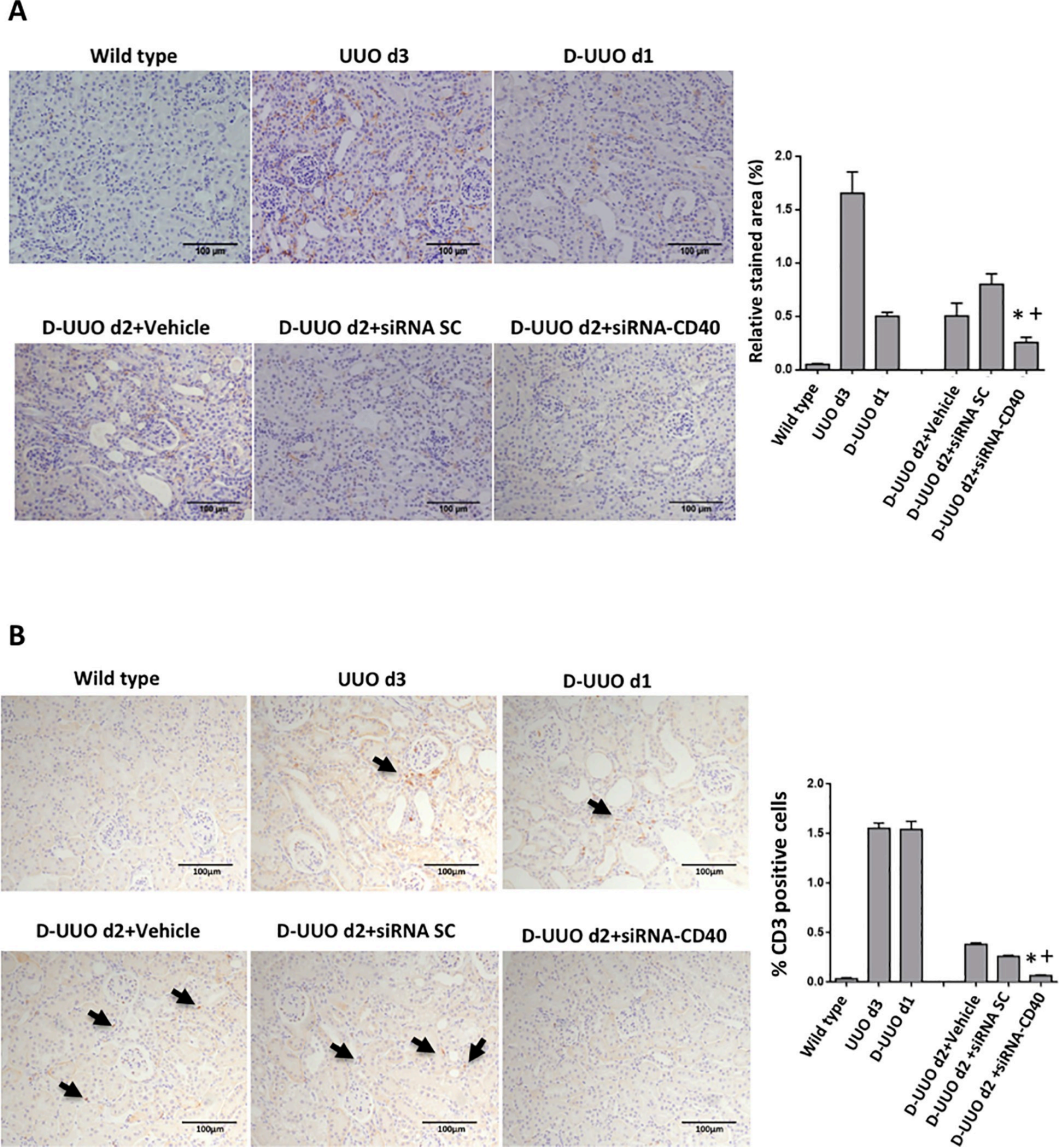
Renal inflammatory cell infiltration in the obstructed kidney. **(A)** Representative images of macrophage interstitial infiltration (F4/80 staining) and (**B)** CD3 positive T cell infiltration. Original magnification 100x. Values are represented in graphical quantification and are obtained as relative stained area (%). Both macrophages and T cells showed a decrease in infiltration after siRNA-CD40. ANOVA test; values are means ± SE, *p ≤ 0.05 vs D-UUO d2+Vehicle, ^+^p ≤ 0.05 vs D-UUO d2+siRNA SC.

Inflammatory cell infiltration evaluated by F4/80 macrophage and CD3 T cell population, was significantly increased at day 3 of obstruction. Once the clamp was removed (D-UUO d1 and D-UUO d2), we observed the amelioration of the inflammatory response. Macrophage interstitial infiltration was significantly reduced in animals treated with siRNA-CD40 (Fig 4A). Furthermore, lymphocytic infiltration was also significantly decreased in siRNA CD40 treated mice compared to SC and vehicle groups (Fig 4B).

### Renal Interstitial fibrosis

**Fig 5:**
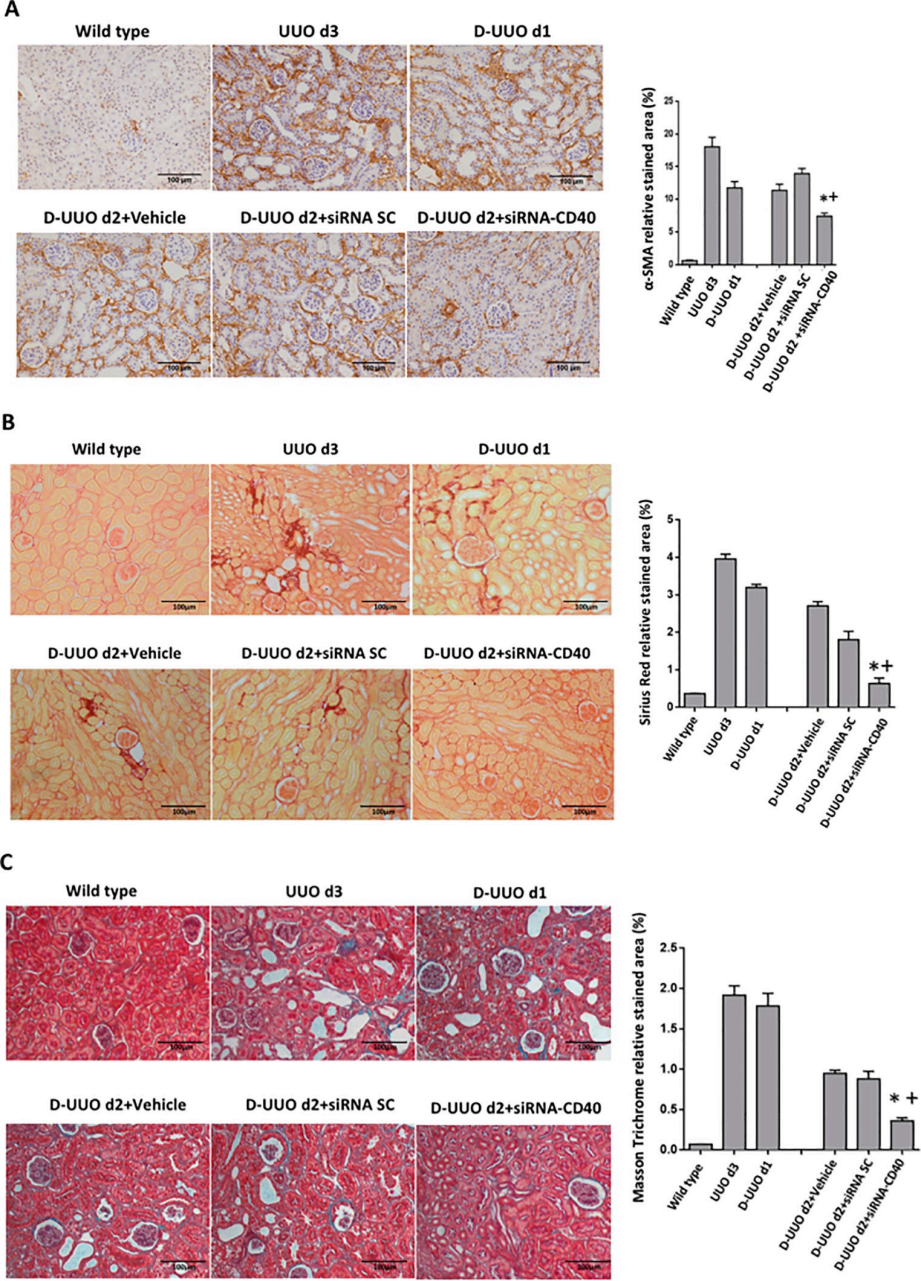
Interstitial fibrosis evaluation of the obstructed kidneys. **(A) Representative images of α-SMA (B) Sirius Red and (C) Masson’s trichrome staining.** Original magnification 100x. Graphical quantification of α-SMA, Sirius Red and Masson’s Trichrome from each group. Values are obtained as relative stained area (%). ANOVA test; values are means ± SE, *p ≤ 0.05 vs D-UUO d2+Vehicle, ^+^p ≤ 0.05 vs D-UUO d2+siRNA SC.

Interstitial fibrosis was observed in the obstructed kidneys at day 3 as shown by collagen deposition in the interstitium analyzed by Sirius Red and Masson Trichrome staining. Moreover, there was widespread expression of α-SMA in the interstitium adjacent to the dilated tubules of the obstructed kidneys, which indicated the presence of interstitial myofibroblasts. After removing the clamp, the collagen deposit and α-SMA-positive signals were significantly decreased. In comparison with the other groups, the administration of siRNA-CD40 significantly reduced the interstitial fibrosis associated with the obstructive uropathy (Fig 5).

### Kidney gene expression

**Fig 6:**
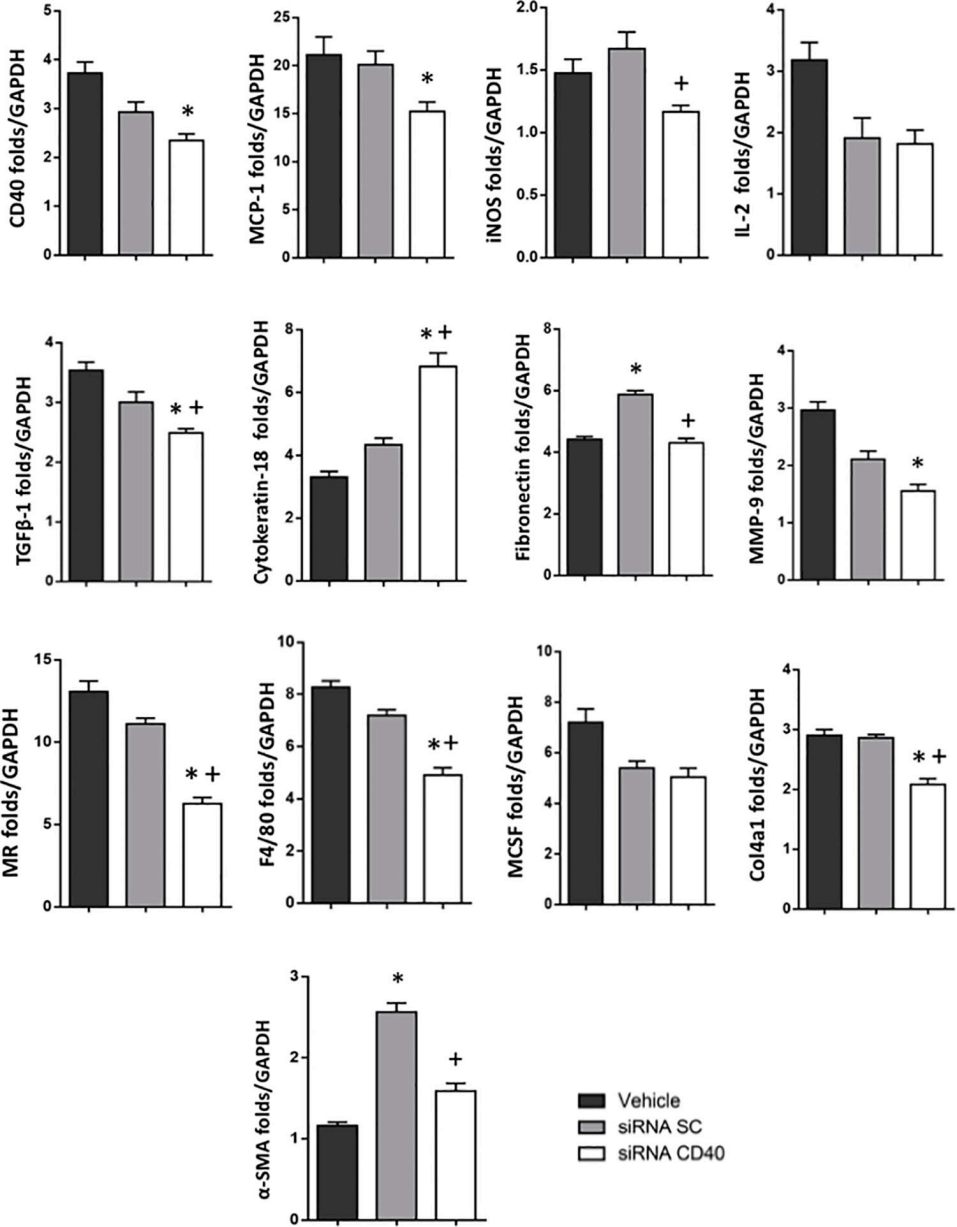
Gene expression profile of the obstructed kidney. For all qRT-PCR analysis, all data are compared to wild type levels. Evaluation of macrophage markers, fibrosis matrix proteins, pro-inflammatory and pro-fibrotic genes after the administration of siRNA-CD40. ANOVA test; values are means ± SE, *p ≤ 0.05 vs D-UUO d2+Vehicle, ^+^p ≤ 0.05 vs D-UUO d2+siRNA SC.

As observed in Fig 6, CD40 mRNA expression was significantly increased in the vehicle group. The administration of siRNA-CD40, as expected, was associated with a significant reduction of renal CD40 mRNA expression compared to the vehicle group. In parallel, pro-inflammatory cytokines such as MCP-1 and iNOS were clearly reduced with the siRNA-CD40 administration. Pro-fibrotic cytokine such TGF-β1, showed significantly lower levels after siRNA-CD40 administration regarding the others groups. Besides, significant reduction of macrophage markers such as F4/80 and Mannose receptor; fibrosis matrix proteins such as Fibronectin, MMP-9, Collagen IV and α-SMA were observed after the administration of siRNA-CD40. Interestingly the cytokeratin-18 was up-regulated with siRNA-CD40 therapy (Fig 6).

## Discussion

In this work, we have investigated the progression of obstructive nephropathy lesions in the D-UUO mouse model in which CD40 expression was silenced “in vivo” with a specific siRNA. Our study demonstrates that siRNA-CD40 ameliorates the renal inflammation and fibrosis induced by UUO renal injury.

CD40, a marker of pro-inflammatory cytokines, plays a crucial role in the onset and maintenance of the inflammatory reaction, and it is expressed by most immune cells as well as by other nonimmune cells such as tubular epithelial cells, where play a critical role in the pathogenesis of renal inflammatory response [21]^,^[22].

Interaction of CD40 with its ligand stimulates downstream signaling and has been targeted in many disorders by using selective pharmacological inhibitors[16]. In this sense, our group has previously demonstrated that a specific CD40 siRNA was effective in reducing CD40 tissue expression[14], while promoting an anti-inflammatory blockade of several transcriptional gene networks in endothelial cells[23].

siRNA are small molecules filtered in the kidney, so kidney disease is a good target to this kind of process. Therapies using siRNA have being developed for various kidney clinical conditions, but few studies have primarily been used to treat obstructive nephropathy[24]. In the present work, UUO increases CD40 expression significantly and a specific siRNA was administrated in a systemic CD40 silencing strategy and the reduction in the inflammation and fibrosis lesions was confirmed. A possible explanation for this powerful anti-inflammatory effect may be an attenuated production of the chemokines and adhesion molecules necessary for interstitial inflammation to occur, since a reduction in the number of plaque-infiltrating F4/80 or macrophages, CD40 cells and cytokines was observed.

In the present study, interstitial macrophage and lymphocyte infiltration, renal tubule dilation, renal myofibroblast expansion, and collagen fiber deposition were all observed. After the release of the obstruction, all these were declined nearly to basal levels. The administration of siRNA-CD40 was associated with a significant reduction of renal CD40 mRNA expression regarding D-UUO and siRNA SC groups. Furthermore, kidney mRNA gene expression analysis showed significantly lower levels of pro-inflammatory and pro-fibrotic cytokines after siRNA-CD40 administration. These results together indicate that there is an increase of the pro-inflammatory and pro-fibrotic environment within the kidney following obstructive injury with subsequent decrease after de-obstruction, and with greater reduction after siRNA-CD40 administration that translates in a significant reduction in the severity of acute renal failure assessed by serum creatinine.

TGF-β1 is considered the most critical factor in the pathogenesis of renal interstitial fibrosis and can be found in a variety of Chronic kidney disease[25]. Moreover, renal interstitial fibroblasts are the major effector cells contributing to EMT (epithelial-mesenchymal transition), collagen is a major component of extracellular matrix, and α-SMA is one of the most important proteins in the EMT process, and its expression in activated EMT would be substantially increased[26,27]. In this experiment, we observed significantly increased TGF-β1 and α-SMA expression in the obstructed kidney, which was more reversed after treatment with siRNA-CD40.

This early reversibility is explained because fibrosis appears on the third day of obstruction and is established at 7 days where remain for longer time[28]. The duration of UUO and the extent of cell loss play a pivotal role in the recovery of GFR (glomerular filtration rate) and subsequent renal remodeling and repair. UUO for >72h may lead to renal fibrotic and apoptotic changes culminating in a permanent decrease in GFR. It have been demonstrated that relief of the obstruction after two to five days permits recovery of renal structure and function[29]. Whereas in this reversible UUO model of three days obstruction no advanced fibrosis changes has been developed yet for the rapid decrease of dynamic factors that develop fibrosis.

Besides, we observed that siRNA-CD40 treatment resulted in decreased macrophage and lymphocyte infiltration into tubulointerstitial areas after renal injury. Macrophage accumulation correlates closely with the progression of renal disease and, importantly, systemic macrophage depletion alleviates renal injury and renal interstitial fibrosis after UUO[17].

Matrix metalloproteinase-9 (MMP-9) was previously thought to be anti-fibrotic due to their ability to induce extracellular matrix remodeling. However, it has also been recognized that is involved in the initiation and progression of kidney fibrosis and it can also exert pro-fibrotic effects under various pathological conditions. The exact mechanism by which MMP-9 contributes to renal fibrosis still is a matter of debate. We have previously reported in a UUO murine model, that MMP-9 expression was significantly reduced when an anti-inflammatory macrophage cell therapy was performed[18]. The MMP-9 result in this article is in line with our previous results but also with those reported by Tan et al suggesting that MMP-9 contributes to the pathogenesis of renal fibrosis in UUO via macrophage recruitment[30].

The apparent discrepancy between maximal damage and normal serum creatinine at day 3 is justified by the presence of the contralateral kidney. At this time, we performed ureter deobstruction and contralateral nephrectomy and therefore all the renal function relied on the recovery of the de-obstructed kidney. This is the reason why the creatinine value peaked at 24h post de-clamping.

The cytokeratin-18 is a specific marker of renal tubular epithelial cell integrity[31]. The administration of siRNA CD40 significantly increased the expression of this cytokeratin-18 in kidney tissue. This result was in line with histologic amelioration and the reduction of several pro-inflammatory and pro-fibrotic genes. These results suggested that mice treated with siRNA CD40 better preserved the structural integrity of their kidney tissue.

The irreversible model of UUO is a most traditional model for renal fibrosis induction but is not focused upon the intrinsic cell biology of the kidney following reversal UUO, so it is not ideal to study this type of treatment[8,9,32]. Moreover, in our study, contrary to others reversible models described[29,33], the contralateral kidney was removed, leaving the previous obstructed kidney as a life sustaining organ, which was very important to test the true efficacy of the siRNA-CD40. In order to discard an unspecific silencing effect, we used and scrambled, non-specific siRNA. This group was similar to vehicle for the majority of results, including renal function, cell infiltration and fibrosis. Indeed, CD40 siRNA provided a significant therapeutic effect in comparison to both vehicle and SC siRNA control groups.

Thus, siRNA-CD40 is a novel signaling pathway mediating the fibrogenic effect delaying the process of CKD and could be a new therapeutic target. These initial results open the door to clinical trials silencing CD40 not only in the UUO inflammatory response, but also in other autoimmune and inflammatory diseases where CD40 have a key role.

In conclusion, the present study demonstrated: First, that the expression of CD40 was inhibited by siRNA-CD40 delivered in a D-UUO mice model with strongest inhibitory efficacy. Second, the administration of siRNA-CD40 therapy reduces the severity of the acute kidney injury induced by obstructive uropathy and promotes kidney repair representing a novel alternative therapeutic strategy. Third, the D-UUO model described here is a simple, well-tolerated, reproducible and more reliable method, since it includes the nephrectomy of the contralateral kidney. Offers to explore the induction and resolution of kidney inflammation together with key aspects of tissue repair post injury at a cellular and molecular level.

## References

[1] Chevalier RL. Pathogenesis of renal injury in obstructive uropathy. Curr Opin Pediatr. 2006;18: 153–160. 10.1097/01.mop.0000193287.56528.a4 16601495

[2] Zeisberg M, Neilson EG. Mechanisms of Tubulointerstitial Fibrosis. J Am Soc Nephrol. 2010;21: 1819–1834. 10.1681/ASN.2010080793 20864689

[3] Zhao J, Wang L, Cao A, Jiang M-Q, Chen X, Wang Y, et al HuangQi Decoction Ameliorates Renal Fibrosis via TGF-β/Smad Signaling Pathway. Cell Physiol Biochem. 2016;38: 1761–1774. 10.1159/000443115 27161221

[4] Chevalier RL. Counterbalance in functional adaptation to ureteral obstruction during development. Pediatr Nephrol. 1990;4: 442–4. Available: http://www.ncbi.nlm.nih.gov/pubmed/2206915 2206915 10.1007/BF00862533

[5] Puri TS, Shakaib MI, Chang A, Mathew L, Olayinka O, Minto AWM, et al Chronic kidney disease induced in mice by reversible unilateral ureteral obstruction is dependent on genetic background. Am J Physiol Renal Physiol. 2010;298: F1024–32. 10.1152/ajprenal.00384.2009 20089676 PMC2853313

[6] Chaabane W, Praddaude F, Buleon M, Jaafar A, Vallet M, Rischmann P, et al Renal functional decline and glomerulotubular injury are arrested but not restored by release of unilateral ureteral obstruction (UUO). Am J Physiol Physiol. 2013;304: F432–F439. 10.1152/ajprenal.00425.2012 23220725

[7] Chevalier RL, Peters CA. Congenital urinary tract obstruction: Proceedings of the State-Of-The-Art Strategic Planning Workshop-National Institutes of Health, Bethesda, Maryland, USA, 11–12 March 2002. Pediatr Nephrol. 2003;18: 576–606. 10.1007/s00467-003-1074-8 12720078

[8] Cochrane AL, Kett MM, Samuel CS, Campanale N V, Anderson WP, Hume DA, et al Renal structural and functional repair in a mouse model of reversal of ureteral obstruction. J Am Soc Nephrol. 2005;16: 3623–30. 10.1681/ASN.2004090771 16221872

[9] Tapmeier TT, Brown KL, Tang Z, Sacks SH, Sheerin NS, Wong W. Reimplantation of the ureter after unilateral ureteral obstruction provides a model that allows functional evaluation. Kidney Int. 2008;73: 885–889. 10.1038/sj.ki.5002797 18200000

[10] Ke B, Shen X-D, Gao F, Tsuchihashi S, Farmer DG, Briscoe D, et al The CD154-CD40 T-cell co-stimulation pathway in liver ischemia and reperfusion inflammatory responses. Transplantation. 2005;79: 1078–83. Available: http://www.ncbi.nlm.nih.gov/pubmed/15880047 15880047 10.1097/01.tp.0000161248.43481.a2 PMC4470618

[11] Hollenbaugh D, Mischel-Petty N, Edwards CP, Simon JC, Denfeld RW, Kiener PA, et al Expression of functional CD40 by vascular endothelial cells. J Exp Med. 1995;182: 33–40. Available: http://www.ncbi.nlm.nih.gov/pubmed/7540655 7540655 10.1084/jem.182.1.33 PMC2192103

[12] de Ramon L, Ripoll E, Merino A, Lúcia M, Aran JM, Pérez-Rentero S, et al CD154-CD40 T-cell co-stimulation pathway is a key mechanism in kidney ischemia-reperfusion injury. Kidney Int. 2015;88: 538–49. 10.1038/ki.2015.146 25993320 PMC4558568

[13] de Ramon L, Jarque M, Ripoll E, Bestard O, Grinyo JM, Torras J. RNAi-Based Therapy in Experimental Ischemia-Reperfusion Injury. The New Targets. Curr Pharm Des. 2016;22: 4651–4657. Available: http://www.ncbi.nlm.nih.gov/pubmed/27510493 27510493 10.2174/1381612822666160719103955

[14] Hueso M, De Ramon L, Navarro E, Ripoll E, Cruzado JM, Grinyo JM, et al Silencing of CD40 in vivo reduces progression of experimental atherogenesis through an NF-κB/miR-125b axis and reveals new potential mediators in the pathogenesis of atherosclerosis. Atherosclerosis. 2016;255: 80–89. 10.1016/j.atherosclerosis.2016.11.002 27835742

[15] Ripoll È, Merino A, Herrero-Fresneda I, Aran JM, Goma M, Bolaños N, et al CD40 gene silencing reduces the progression of experimental lupus nephritis modulating local milieu and systemic mechanisms. Zirlik A, editor. PLoS One. 2013;8: e65068 10.1371/journal.pone.0065068 23799000 PMC3683035

[16] Zhang B, Wu T, Chen M, Zhou Y, Yi D, Guo R. The CD40/CD40L system: A new therapeutic target for disease. Immunol Lett. 2013;153: 58–61. 10.1016/j.imlet.2013.07.005 23892087

[17] Guiteras R, Flaquer M, Cruzado JM. Macrophage in chronic kidney disease. Clin Kidney J. 2016;9: 765–771. 10.1093/ckj/sfw096 27994852 PMC5162417

[18] Guiteras R, Sola A, Flaquer M, Hotter G, Torras J, Grinyó JM, et al Macrophage Overexpressing NGAL Ameliorated Kidney Fibrosis in the UUO Mice Model. Cell Physiol Biochem. 2017;42: 1945–1960. 10.1159/000479835 28793288

[19] Ripoll E, Pluvinet R, Torras J, Olivar R, Vidal A, Franquesa M, et al In vivo therapeutic efficacy of intra-renal CD40 silencing in a model of humoral acute rejection. Gene Ther. 2011;18: 945–952. 10.1038/gt.2011.39 21472009

[20] Flaquer M, Franquesa M, Vidal A, Bolaños N, Torras J, Lloberas N, et al Hepatocyte growth factor gene therapy enhances infiltration of macrophages and may induce kidney repair in db/db mice as a model of diabetes. Diabetologia. 2012;55: 2059–2068. 10.1007/s00125-012-2535-z 22460762 PMC3369134

[21] Elgueta R, Benson MJ, de Vries VC, Wasiuk A, Guo Y, Noelle RJ. Molecular mechanism and function of CD40/CD40L engagement in the immune system. Immunol Rev. 2009;229: 152–172. 10.1111/j.1600-065X.2009.00782.x 19426221 PMC3826168

[22] van Kooten C, Woltman AM, Daha MR. Immunological function of tubular epithelial cells: the functional implications of CD40 expression. Exp Nephrol. 2000;8: 203–7. 10.1159/000020669 10940717

[23] Pluvinet R, Olivar R, Krupinski J, Herrero-Fresneda I, Luque A, Torras J, et al CD40: an upstream master switch for endothelial cell activation uncovered by RNAi-coupled transcriptional profiling. Blood. 2008;112: 3624–37. 10.1182/blood-2008-03-143305 18669876

[24] Yang C, Nilsson L, Cheema MU, Wang Y, Frøkiær J, Gao S, et al Chitosan/siRNA Nanoparticles Targeting Cyclooxygenase Type 2 Attenuate Unilateral Ureteral Obstruction-induced Kidney Injury in Mice. Theranostics. 2015;5: 110–123. 10.7150/thno.9717 25553102 PMC4278998

[25] Yuan A, Lee Y, Choi U, Moeckel G, Karihaloo A. Chemokine receptor Cxcr4 contributes to kidney fibrosis via multiple effectors. Am J Physiol Renal Physiol. 2015;308: F459–72. 10.1152/ajprenal.00146.2014 25537742 PMC4346747

[26] Burns WC, Kantharidis P, Thomas MC. The Role of Tubular Epithelial-Mesenchymal Transition in Progressive Kidney Disease. Cells Tissues Organs. 2007;185: 222–231. 10.1159/000101323 17587828

[27] Shing LK, Meng X-M, Nikolic-Paterson DJ, Lan HY. Inflammatory processes in renal fibrosis. Nat Publ Gr. 2014; 10.1038/nrneph.2014.114 24981817

[28] Chevalier RL, Forbes MS, Thornhill BA. Ureteral obstruction as a model of renal interstitial fibrosis and obstructive nephropathy. Kidney International. 2009 10.1038/ki.2009.86 19340094

[29] Chevalier RL, Thornhill BA, Chang AY, Cachat F, Lackey A. Recovery from release of ureteral obstruction in the rat: Relationship to nephrogenesis. Kidney Int. 2002;61: 2033–2043. 10.1046/j.1523-1755.2002.00359.x 12028444

[30] Kui Tan T, Zheng G, Hsu T-T, Ra Lee S, Zhang J, Zhao Y, et al Matrix metalloproteinase-9 of tubular and macrophage origin contributes to the pathogenesis of renal fibrosis via macrophage recruitment through osteopontin cleavage. Lab Investig. 2013;93: 434–449. 10.1038/labinvest.2013.3 23358111

[31] Snider NT. Kidney keratins: cytoskeletal stress responders with biomarker potential. Kidney Int. 2016;89: 738–740. 10.1016/j.kint.2015.12.040 26994569

[32] Chaabane W, Praddaude F, Buleon M, Jaafar A, Vallet M, Rischmann P, et al Renal functional decline and glomerulotubular injury are arrested but not restored by release of unilateral ureteral obstruction (UUO). Am J Physiol Physiol. 2013; 10.1152/ajprenal.00425.2012 23220725

[33] Cochrane AL. Renal Structural and Functional Repair in a Mouse Model of Reversal of Ureteral Obstruction. J Am Soc Nephrol. 2005; 10.1681/ASN.2004090771 16221872

